# Isolated revision meniscal repair – failure rates, clinical outcome, and patient satisfaction

**DOI:** 10.1186/s12891-018-2368-0

**Published:** 2018-12-21

**Authors:** Andreas Fuchs, Ferdinand Kloos, Gerrit Bode, Kaywan Izadpanah, Norbert P. Südkamp, Matthias J. Feucht

**Affiliations:** 1 Department of Orthopedic Surgery and Traumatology, Freiburg University Hospital, Albert Ludwigs University Freiburg, Hugstetter Straße 55, 79106 Freiburg, Germany; 2 Department of Orthopaedic Sports Medicine, Technical University Munich, Ismaninger Straße 22, 81675 Munich, Germany

**Keywords:** Meniscus, Failure, Revision, Knee, Arthroscopy

## Abstract

**Background:**

Failure of isolated primary meniscal repair must be expected in approximately 10–25% of cases. Patients requiring revision surgery may benefit from revision meniscal repair, however, the results of this procedure remain underreported. The purpose of this study was therefore to evaluate the outcome and failure rates of isolated revision meniscal repair in patients with re-tears or failed healing after previous meniscal repair in stable knee joints.

**Methods:**

A chart review was performed to identify all patients undergoing revision meniscal repair between 08/2010 and 02/2016. Only patients without concomitant procedures, without ligamentous insufficiency, and a minimum follow-up of 24 months were included. The records of all patients were reviewed to collect patient demographics, injury patterns of the meniscus, and details about primary and revision surgery. Follow-up evaluation included failure rates, clinical outcome scores (Lysholm Score, KOOS Score), sporting activity (Tegner scale), and patient satisfaction.

**Results:**

A total of 12 patients with a mean age of 22 ± 5 years were included. The mean time between primary repair and revision repair was 27 ± 21 months. Reasons for failed primary repairs were traumatic re-tears in 10 patients (83%) and failed healing in two patients (17%). The mean follow-up period after revision meniscal repair was 43 (± 23.4) months. Failure of revision meniscal repair occurred in 3 patients (25%). In two of these patients, successful re-revision repair was performed. At final follow-up, the mean Lysholm Score was 95.2 (± 4.2) with a range of 90–100, representing a good to excellent result in all patients. The final assessment of the KOOS subscores also showed good to excellent results. The mean Tegner scale was 6.8 ± 1.8, indicating a relatively high level of sports participation. Ten patients (83%) were either satisfied or very satisfied with the outcome.

**Conclusion:**

In patients with re-tears or failed healing after previous isolated meniscal repair, revision meniscal repair results in good to excellent knee function, high level of sports participation, and high patient satisfaction. The failure rate is slightly higher compared to isolated primary meniscal repair, but still acceptable. Therefore, revision meniscal repair is worthwhile in selected cases in order to save as much meniscal tissue as possible.

## Background

The menisci of the knee joint play an important role in load transmission, shock absorption, proprioception, cartilage lubrication, and joint stability [1]. Therefore, integrity of the menisci is crucial for long term knee function. For several decades, total or partial meniscectomy was the treatment of choice for symptomatic meniscus tears [2]. However, with the growing understanding of meniscal function and an increasing number of studies reporting suboptimal results after meniscectomy [3] meniscal repair has become widely accepted [4]. Compared to partial meniscectomy, meniscus repair has shown to result in better knee function, higher activity levels, less progression of osteoarthritis, and cost saving [5–9]. However, the reoperation rate after meniscal repair is higher [5] and according to recent systematic reviews, a failure rate of approximately 11–23% must be excepted [10–12]. With the increasing numbers of meniscal repairs performed during the last decade [13], the number of failed meniscus repairs will also increase. Until now, meniscectomy seems to be the preferred technique to address failed meniscus repairs [14–17]. However, revision meniscal repair may provide better long-term outcomes than meniscectomy, but the results of this procedure remain underreported [18–21]. It is therefore unclear if revision meniscal repair is worthwhile.

The purpose of this study was to evaluate the clinical outcome and failure rates of isolated revision meniscal repair in patients with re-tears or failed healing after previous meniscal repair in stable knee joints. We hypothesized that revision meniscal repair would demonstrate good clinical outcomes and an acceptable failure rate.

## Methods

### Study design and patient selection

A retrospective case series was conducted to study the clinical outcome and failure rates of isolated revision meniscal repair in patients with re-tears or failed hailing after previous isolated meniscal repair in stable knee joints at our institution. The study was approved by the institutional review board, and the study was performed in accordance with the Declaration of Helsinki. Informed consent was obtained from all patients.

A chart review was performed using our electronic medical record system to identify all patients undergoing meniscal repair between 2010 and 2016. For further patient selection, only patients without concomitant procedures and without ligamentous insufficiency were included.

A total of 196 patients were finally identified. Of those, 31 patients presented to our clinic with a symptomatic re-tear or failed healing as determined by clinical examination and magnetic resonance imaging (MRI). All patients underwent revision surgery. Partial meniscectomy was performed in 19 patients and 12 patients underwent isolated revision meniscal repair. Revision meniscal repair was indicated for unstable tears located in the red-red or red-white zone and good tissue quality. Partial meniscectomy was indicated in patients with meniscal tears located in the white-white zone, severely degenerated meniscal tissue, and tears which could technically not be stabilized.

For the purpose of this study, only patients after isolated revision meniscal repair with a minimum follow-up of 24 months were included. The records of all patients were reviewed to collect patient demographics, injury patterns of the meniscus, and details about primary and revision surgery.

### Surgical technique and postoperative rehabilitation

Revision meniscal repair was performed using a routine arthroscopic setup. A probe was used to confirm the preoperative diagnosis and characterize the meniscus tear. Suture material from the previous repair was removed if necessary and the tear side was debrided with a shaver or rasp. Depending on the tear morphology and localization, revision repair was performed with either an all-inside technique (FasT-Fix®, Smith & Nephew, Andover, USA, or Meniscal Viper®, Arthrex, Naples, USA), an inside-out technique (SharpShooter®, Ivy Sports Medicine, Montvale, USA), or the combination of both. A combination of horizontal and vertical suture configuration was used and the number of sutures depended on the tear size. In general, sutures were added until the tear was considered stable by probing. Root tears were repaired using a transtibial pull-out suture technique.

Postoperatively, patients were restricted to partial weight bearing (20 kg) for 6 weeks, followed by gradual increase of weightbearing over the following 2–4 weeks. During the first 6 weeks, flexion was limited to 90°. Full squatting was permitted 4 months after surgery and return to sporting activities was allowed after 6 months.

### Follow-up evaluation

Failure of revision meniscal repair was defined as repeat surgery on the same meniscus during the follow-up period or meniscal symptoms such as pain, catching, or locking. To assess the functional outcome, the Lysholm score and Knee Injury and Osteoarthritis Outcome Score (KOOS) were used. Sporting activity was assessed with the Tegner activity scale. Patients satisfaction with revision meniscal repair was assessed by asking the patients if they were very satisfied, satisfied, partially satisfied, or dissatisfied. Furthermore, the patients were asked if they would undergo the surgery again. In patients who failed revision meniscal repair and underwent subsequent surgery, follow-up evaluation was conducted after re-revision surgery.

## Results

### Patient demographics and surgical details

A total of 12 patients were included. The mean patient age at revision meniscal repair was 22 ± 5 years (range, 17–34 years) and the mean body mass index was 22.3 ± 2.6 kg/m^2^ (range, 19.0–29.3 kg/m^2^). Nine patients were male (75%) and the left knee was affected in 8 patients (67%).

Injury specific information and surgical details of each patient are provided in Table 1.

**Table 1:** Injury specific information and surgical details

Patient No.	Time between primary and revision repair (ms)	Reason for revision meniscal repair	Tear location	Tear zone	Tear type at primary repair	Tear type at revision repair	Suture technique at primary repair	Suture technique at revision repair
1	56	Traumatic re-tear	Medial meniscus (Anterior horn + midbody + posterior horn)	Red-white	BH	BH	I-O (4x)	I-O (5x) A-I (1x)
2	8	Traumatic re-tear	Medial meniscus (midbody + posterior horn)	Red-white	BH	LT	I-O (2x)	I-O (1x) A-I (1x)
3	35	Traumatic re-tear	Medial meniscus (Anterior horn + midbody + posterior horn)	Red-white	LT	BH	I-O (2x)	I-O (3x)
4	21	Traumatic re-tear	Medial meniscus (Anterior horn + midbody + posterior horn)	Red-white	LT	VT	I-O (1x)	I-O (5x) A-I (1x)
5	48	Traumatic re-tear	Medial meniscus (midbody + posterior horn)	Red-red	LT	LT	I-O (2x)	I-O (2 x)
6	28	Traumatic re-tear	Lateral meniscus (posterior horn)	Red-white	RT	CT	T (2x)	A-I (2x)
7	3	Failed healing	Lateral meniscus (posterior horn)	Red-red	RT	RT	T (2x)	T (1x)
8	4	Traumatic re-tear	Medial meniscus (midbody + posterior horn)	Red-white	BH	CT	I-O (2x)	A-I (2x) I-O (2x)
9	27	Failed healing	Medial meniscus (midbody)	Red-white	BH	BH	I-O (3x) A-I (1x)	I-O (3x)
10	70	Traumatic re-tear	Lateral meniscus (midbody + posterior horn)	Red-white	LT	CT	A-I (1x)	A-I (3x)
11	10	Traumatic re-tear	Medial meniscus (midbody + posterior horn)	Red-white	LT	VT	A-I (1x)	A-I (1x)
12	9	Traumatic re-tear	Lateral meniscus (posterior horn)	Red-red	BH	LT	A-I (4x)	A-I (1x)

Abbreviations: *Ms* months, *BH* bucket handle, *LT* longitudinal tear, *RT* root tear, *VT* vertical tear, *CT* complex tear, *I-O* inside-out, *A-I* all-inside, *T* transosseus pull-out

In 10 patients (83%), a traumatic re-tear was the reason for revision meniscal repair. Traumatic re-tears occurred during sports in 8 patients (67%) and during activities of daily living in two patients (17%). Failed healing (biologic failure) was considered the main reason for revision repair in two patients (17%). The mean time between primary repair and revision meniscal repair was 26.6 ± 21 months (range, 3–70 months). The re-tear involved the primary repair site in all cases. The medial meniscus was affected in 8 patients (67%) and the lateral meniscus in 4 patients (33%). All tears were located in the red-red (3 patients, 25%) or red-white zone (9 patients, 75%). At the time of the primary repair, 5 patients (42%) had displaced bucket-handle tears, 5 patients (42%) had longitudinal tears, and two patients (17%) had root tears. At the time of revision repair, 3 patients (25%) had displaced bucket-handle tears, 3 patients (25%) had longitudinal tears, 3 patients (25%) had complex tears, 2 patients (17%) had vertical tears, and one patient (8%) had a root tear. The tear type changed between primary and revision repair in 7 cases (58%; cases 2, 3, 4, 6, 8, 11, and 12).Revision meniscal repair was performed using an inside-out technique in 3 patients (25%), an all-inside technique in 4 patients (34%), a combination of inside-out and all-inside techniques in 4 patients (34%), and a transtibial pullout suture technique in 1 patient (8%). The mean number of sutures was 2.4 ± 1.2 (range, 1–4) at primary repair and 2.8 ± 1.7 (range, 1–6) at revision repair. Compared to primary repair, the number of sutures required for revision repair increased in 5 patients (42%), remained unchanged in 4 patients (33%), and decreased in 3 patients (25%).

### Failure rates, clinical results, and patient satisfaction

All patients were available for follow-up evaluation. The mean follow-up period after revision meniscal repair was 43 ± 20.4 months (range, 24–78 months). Failure of revision meniscal repair occurred in 3 patients (case 2, 3, and 5), therefore, the overall failure rate was 25%. In two of these patients, re-revision repair was performed (case 3 and 5), whereas another patient underwent partial meniscectomy (case 2). The follow-up period of both patients undergoing re-revision repair was more than 24 months after the last operation. At final follow-up, both patient had no meniscus-specific symptoms and excellent functional scores. Therefore, the combined success rate of revision and re-revision repair was 92% (11 of 12 cases).

The detailed outcomes of each patient are shown in Table 2. Ten patients (83%) were either very satisfied or satisfied with the outcome. Only two patients (17%) were partially satisfied with the outcome, and only two patients stated that they would not undergo the same surgery again. In both patients, failure of revision repair occurred. The mean Tegner scale was 6.8 ± 1.8 (range, 4–10), indicating a relatively high level of sports participation. The mean Lysholm Score was 95.2 ± 4.2 with a range of 90–100, representing a good to excellent result in all patients. The final assessment of the KOOS subscores also showed good to excellent results with the following mean values: KOOS symptoms 95.5 ± 4.4 (range, 85.7–100), KOSS pain 97.0 ± 3.1 (range, 94.4–100), KOOS ADL 99.5 ± 1.1 (range, 96.9–100), KOOS Sport/Rec 92.5 ± 10.3 (70–100), KOOS QOL 81.8 ± 12.1 (range, 56,3–100).

**Table 2:** Outcome of revision meniscal repair in each patient

Patient No.	Failure of revision meniscal repair	Satisfaction	Tegner scale	Lysholm Score	KOOS symptoms	KOOS pain	KOOS ADL	KOOS sport/rec	KOOS QOL
1	No	satisfied	7	99	96	97	100	80	81
2	Yes^b^	partially satisfied	4	90	86	94	100	70	88
3	Yes^a^	partially satisfied	6	100	96.4	92	100	100	56
4	No	satisfied	5	90	92.9	97	97	100	67
5	Yes^a^	very satisfied	6	99	100	100	100	85	100
6	No	very satisfied	10	95	96	100	100	100	94
7	No	very satisfied	7	100	100	100	100	100	88
8	No	very satisfied	8	90	89	97	100	100	88
9	No	very satisfied	9	90	96	94	97	100	81
10	No	satisfied	5	94	93	92	100	80	69
11	No	very satisfied	9	100	100	100	100	100	94
12	No	very satisfied	5	95	100	100	100	95	75

Abbreviations: *KOOS* Knee Injury and Osteoarthritis Outcome score, *ADL* activities of daily living; sport/rec, sport and recreation, *QOL* quality of life

^a^Both patients underwent subsequent re-revision meniscal repair. Outcome scores were obtained > 24 months after re-revision repair

^b^The patient underwent subsequent partial meniscectomy. Outcome scores were obtained > 24 months after partial meniscectomy

## Discussion

The most important findings of this study were that isolated revision meniscal repair results in good to excellent knee function, high level of sports participation, and high patient satisfaction in patients with re-tears or failed healing after previous isolated meniscal repair. The failure rate of 25% is slightly higher compared to isolated primary meniscal repair, but still acceptable. Therefore, revision meniscal repair seems to be a valuable treatment option in selected cases.

Integrity of the menisci is vital to maintain knee joint health, and the management of meniscal tears has evolved over the last decades [2]. Current evidence suggests that meniscus repair should be preferred over meniscectomy whenever possible in order to preserve as much meniscus tissue as possible [5–8]. However, failure of primary meniscal repairs must be expected in approximately 11–23% of patients [10–12]. Kurosaka et al. demonstrated that repaired menisci may tear again even after arthroscopically confirmed healing [22]. In their study, stable healing of the repaired meniscus was observed in 90 of 111 patients during second-look arthroscopies at a mean of 13 months after repair. Of the 90 patients with stable repairs, however, 13 patients sustained a re-tear of the meniscus, which was always located at the primary repair site. This finding corresponds well to the findings of our study. Most of our patients sustained a traumatic re-tear during sporting activities. Given the fact that most patients were able to perform sports after the primary repair and the relatively long time period between primary repair and revision repair, it can be hypothesized that healing occurred after primary meniscal repair. Similarly to the findings of Kurosaka et al., the re-tear in our series occurred at the primary repair site in all cases [22]. This may be attributed to the mechanically less stable scar tissue at the repair side observed in animal studies [23, 24].

With the increasing numbers of meniscal repairs observed during the last decade [13], failed repairs will become a more common problem. Therefore, recommendations for appropriate treatment of these cases will become more important. Similarly to primary meniscal repair, revision meniscal repair may provide better functional outcomes and less progression of osteoarthritis than meniscectomy [5–8]. However, meniscectomy seems to be the preferred treatment option since results of revision meniscal repair remain underreported [14–17]. To the best of our knowledge, only three studies have specifically analysed the results of revision meniscal repair so far [18–20]. Despite differences in patient population, meniscal tear patterns, concomitant procedures, and follow-up period, the results of these studies are generally comparable to our results (Table 3): Voloshin et al. reported on a series of 18 repeat meniscal repairs performed over an 11-year period. After a mean follow-up of 7 years, the clinical success rate was 72% [20]. Imade et al. analyzed the results of revision meniscal repair in 15 patients. The success rate was 67%, and all failed revision repairs had degeneration of the meniscal tissue. The authors therefore concluded that revision meniscal repair should be considered in the setting of a re-torn meniscus without degenerative changes [18]. Krych et al. retrospectively investigated 34 patients at 2 to 17 years after revision meniscal repair. In 13 of these cases, concomitant ACL reconstruction was performed. The clinical success rate was 79%. Younger age was associated with an increased risk of revision repair failure, whereas tear zone, tear pattern, meniscal side, surgical technique, and combined ligament reconstruction were not significant predictors of failure [19]. The main difference between the present and the above stated studies is that we did not include patients who underwent concomitant reconstructive procedures, especially ACL reconstruction. It has been demonstrated that concomitant ACL reconstruction at the time of meniscal repair improves healing rates of the repaired meniscus [14]. We therefore excluded patients with concomitant procedures in order to reduce confounding factors. The present study is therefore the first to focus on isolated revision meniscal repair in stable knee joints.

**Table 3:** Outcome of revision meniscal repair as reported in the literature

Study	Number of patients	Follow-up period	Failure rate	Lysholm Score	IKDC Score	Tegner Scale
Imade et al., 2014	15	41 months (range, 24–74 months)	33%	97.4 (range, 90–100)	–	5.9 (range, 2–9)
Krych et al., 2016	34	72 months (range, 2 to 17 years)	21%	–	84.8 (range, 51.7–100)	6.2 (range, 3–9)
Voloshin et al., 2003	18	7.33 years (range, 3.25 to 13.75 years)	28%	82.1 (range, 38–100)	–	5.6 (range, 2–8)
Present study	12	43 months (range, 24–78 months)	25%	95.2 (range, 90–100)	–	6.8 (range, 4–10)

Revision meniscal repair seems to have slightly higher failure rates compared to primary repairs. Nepple et al. [10] performed a systematic review of studies reporting the outcome of primary meniscal repair at a minimum of five years postoperatively. A total of 566 knees were included and the pooled rate of meniscal repair failure was 23%. Grant et al. [12] conducted a systematic review to compare the effectiveness of inside-out and all-inside repair techniques for isolated unstable peripheral longitudinal meniscal tears. The rate of clinical failure was 17% for inside-out repairs and 19% for all-inside repairs. In a more recent systematic review, Fillingham et al. [11] analyzed the results of inside-out and modern all-inside repairs for isolated meniscal tears. The overall clinical failure rate was 11% with no statistically significant differences between the two techniques. In the present study, the failure rate after isolated revision meniscal repair was 25%, which is slightly higher compared to the above reported failure rates after primary repairs. Furthermore, in 2 of the 3 failures, re-revision repair was performed, with clinically success in both patients. Therefore, the meniscus could be saved in a total of 92% of our patient cohort.

Despite a slightly higher failure rate, the clinical results observed in the present study are comparable to those reported after primary repairs. In the systematic review of Fillingham et al. [11], the mean Lysholm score was 89.0 and the mean Tegner score was 5.7. In the systematic review of Grant et al. [12], the Lysholm score was 90.2 for all-inside repairs and 87.8 for inside-out repairs. Corresponding Tegner scores were 5.5 and 5.6. In our study, the mean Lysholm Score was 95.2 and the mean Tegner score was 6.8. We therefore conclude that revision meniscal repair can achieve the same clinical results and same activity level as primary repair.

This study has several limitations. First, the study population was relatively small, which limits the overall validity of our results. However, revision meniscal repair is performed only infrequently and only patients without concomitant procedures were included. Therefore, our study cohort represents a relatively homogenous collective with regard to surgical treatment. Second, failure of revision meniscal repair was not evaluated by MRI or second-look arthroscopy. Therefore, the actual failure rate may be underestimated. Third, no statistical analysis was performed because preoperative scores were not available. However, the main intention of this study was to analyze the failure rate and clinical outcome at a minimum of 2 years after isolated revision meniscal repair. Fourth, no control group was analyzed. The present study cannot answer the question whether revision meniscal repair is superior compared to conservative treatment or partial meniscectomy. Further studies are necessary to evaluate whether revision meniscal repair can prevent or delay osteoarthritis.

## Conclusions

In patients with re-tears or failed healing after previous isolated meniscal repair, revision meniscal repair results in good to excellent knee function, high level of sports participation, and high patient satisfaction. Clinical outcome scores and activity level after revision repair are not inferior compared to primary repairs. The failure rate is slightly higher compared to isolated primary meniscal repair, but still acceptable. Therefore, revision meniscal repair is worthwhile in selected cases in order to save as much meniscal tissue as possible.
